# Dads make a difference: an exploratory study of paternal support for breastfeeding in Perth, Western Australia

**DOI:** 10.1186/1746-4358-4-15

**Published:** 2009-11-29

**Authors:** Jenny Tohotoa, Bruce Maycock, Yvonne L Hauck, Peter Howat, Sharyn Burns, Colin W Binns

**Affiliations:** 1 School of Public Health, Curtin Health Innovation Research Institute, Curtin University of Technology, Perth, Western Australia, Australia; 2 School of Population Health, University of Western Australia, Perth, Western Australia, Australia; 3 Centre for Behavioural Research Cancer Control, Curtin University, Perth, Western Australia, Australia

## Abstract

**Background:**

The ability to breastfeed and continue the practice requires dedication, commitment, persistence and support. Mothers often need to overcome many obstacles to successfully breastfeed their babies and maintain their balance of home, family and work commitments. Evidence suggests that fathers want to be involved and be part of the parenthood process, including infant feeding. The role transition from couple to family poses challenges to both parents. Sharing the experience of childbirth and supporting each other in the subsequent infant feeding practices is one of those challenges.

**Methods:**

A qualitative exploratory design was chosen to identify parents' perceptions of what constitutes support for breastfeeding, particularly focusing upon paternal support. Focus groups were conducted with mothers and a focus group, interviews and an online survey were developed for fathers. Thematic analysis was used to identify the main themes.

**Results:**

From a total of 76 participants, the major theme emerging from mothers' data identified that "Dads do make a difference". Three sub-themes included: Anticipating needs and getting the job done; Encouragement to do your best; and Paternal determination and commitment, associated with effective partner support. "Wanting to be involved" was identified from fathers' data as the major theme around their needs. Three sub-themes included: Wanting more information; Learning the role; and Being an advocate.

**Conclusion:**

Sharing the experience of childbirth and supporting each other in the subsequent infant feeding practices was perceived as the best outcome for the majority of new mothers and fathers. Paternal emotional, practical and physical supports were identified as important factors to promote successful breastfeeding and to enrich the experience for the mother and subsequently the father.

**Trail Regristration:**

Australia and New Zealand Clinical Trials Registry: ACTRN12609000667213.

## Background

Breastfeeding is an important strategy in the promotion of child health [1]. Support from others, especially from fathers, is a major factor affecting breastfeeding success [2]. Although breastfeeding initiation rates in Australia are high, with more than 83% of women leaving the hospital breastfeeding, only 23% of infants receive any breast milk by 12 months postpartum [3], which falls short of the international guidelines for infant nutrition [4,5]. Challenges that influence the duration of breastfeeding include community attitudes to breastfeeding [6]. The World Health Organisation (WHO) and the United Nations Children's Fund (UNICEF) both recommend exclusive breastfeeding until six months of age. It is recommended that breastfeeding continue for at least 12 months, and thereafter for as long as mutually desired [5,7]. Breastfeeding trends in Australia have remained largely unchanged over the past decade, but a greater disparity in prevalence rates between lower socioeconomic and higher socioeconomic has been found [8].

Human milk is a bodily fluid which, apart from being an excellent nutritional source for the growing infant, also contains a variety of immune components such as antibodies, growth factors, cytokines, antimicrobial compounds, and specific immune cells [9]. These help to support the immature immune system of the newborn baby, and offer protection from infectious risks during the postnatal period while the immune system matures [9]. Infant formula does not provide protection against viruses or pathogenic bacterial organisms [9]. Despite current scientific evidence that artificial feeding can be a harmful practice, acceptance of breastfeeding as the normal or "default" method of infant feeding remains elusive in the industrialised world [10]. Two global strategies to address the issues of infant formula include the International Code of Marketing of Breast milk Substitutes proposed by WHO in 1981 [11] and the Global Strategy for Infant and Young Child Feeding [12,13] which underpins the Baby-Friendly Hospital Initiative. In Western Australia there are only three Baby-Friendly hospitals amongst a total of 12 maternity hospitals [14]. This lack of proactive breastfeeding infrastructure may contribute to lower breastfeeding duration [15], although Bartington et al. found there was no increase in duration of breastfeeding in a baby friendly hospital setting [16].

Mothers who breastfeed have reduced risk of ovarian cancer, breast cancer and better weight regulation [17-19]. Breastfeeding can assist in attachment development and increase maternal sensitivity [20], and understanding the importance of breastfeeding and the benefits it affords to both the baby and the mother can increase the opportunity for fathers to support their partners in their effort to breastfeed. Sherriff et al. suggest that the father of the baby is one of the most influential persons to the mother, and that they can act as either key supporters or deterrents to breastfeeding [21]. There is strong evidence that fathers can influence the initiation and maintenance of breastfeeding [22,23], contribute to maternal breastfeeding confidence [24-27], influence decisions regarding duration and weaning [28], and that without fathers' support mothers are more likely to breastfeed for a shorter duration [29,30]. Bar-Yam and Darby found that fathers influenced the breastfeeding decision, assistance at first feeding, duration of breastfeeding, and risk factors for bottle feeding [31]. They suggested that more research is needed to identify the methods and means of support that fathers can give their partners to ensure breastfeeding continues for the recommended six months. In addition, when fathers are not able to be supportive, breastfeeding rates were lower [32].

Fathers' involvement in parenting is associated with positive cognitive, developmental, and socio-behavioural child outcomes such as improved weight gain in preterm infants, improved breastfeeding rates, higher receptive language skills, and higher academic achievement [33]. However, Giugliani et al. suggest that fathers need to be better prepared to assume their new role as breastfeeding supporters [34].

Research with mothers identifies fathers as a primary source of support, yet little is known about the nature of this support [35]. This paper identifies what women and men perceive as essential paternal support to facilitate successful breastfeeding. In addition, it ascertains what men believe they need to assist them to be an effective breastfeeding advocate.

## Methods

A qualitative exploratory design was employed to identify the paternal support women wanted to assist them with their breastfeeding. Focus groups were used to identify trends and patterns in perceptions of breastfeeding support. The focus groups aimed to establish the social context about breastfeeding support and enabled interactive discussions to take place among participants. Eight focus groups were conducted with postnatal mothers from a range of socioeconomic backgrounds, with groups comprising four to ten mothers who were breastfeeding or who had breastfed in the past six months. The objectives of these focus groups was to obtain descriptions of the perceived nature of fathers' positive support and to explore the effective support strategies fathers can use to be breastfeeding advocates.

Several attempts were made to recruit fathers from both higher and lower socioeconomic backgrounds for focus groups, but with limited success. Accessing these men proved to be much more challenging than anticipated, although they identified they would respond to a range of flexible data collection processes. A combination of data collection techniques including a focus group, an online survey and telephone interviews were used. Survey Monkey is a web-based survey collector with encrypted responses for confidentiality, and was used to develop and implement the online survey for fathers [36]. The questions in the online survey were identical to those asked in the focus group and in the phone interviews. Two telephone interviews were conducted with fathers who did not have access to the internet and were not available to attend a focus group. The objectives of these data collection methods were to identify fathers' perceptions about breastfeeding; identify factors that encourage fathers to support their partner's breastfeeding (facilitators), and the factors that discourage fathers from supporting their partners' breastfeeding (barriers).

Ethics approval was obtained from Curtin University of Technology and met the conditions of the Helsinki Declaration. This paper is reporting on the formative research conducted in preparation for a randomized controlled trial. Informed consent was obtained from all participants through the provision of an information letter and signed consent forms.

### Recruitment

Purposive sampling of mothers and fathers with breastfeeding infants was conducted over six months from June 2007 to December 2007 across the Perth metropolitan area. Perth is the capital of Western Australia with a population of around 1.2 million people, and has a diverse cultural and ethnic population. Criteria for inclusion were that participants had to be English-speaking, and a mother or father of a breastfed infant. A total of 48 women and 28 men (n = 76) confirmed their willingness to participate. Focus group participants were recruited across several settings via posters inviting participation in the study, at child health clinics, child care centres and early learning centres, to obtain women with a range of experience, socioeconomic levels and occupations. Participants who enquired in response to the advertising strategies were contacted via email or telephone outlining the nature of the study, a choice of times for the focus groups, and the location, along with details about ethical issues such as confidentiality and storage of data. Fathers were primarily recruited by their partners via "wanted" notices placed in early learning centres and child health centres.

### Focus group procedures

The focus groups were conducted at private homes and community centres as nominated by the participants. The focus group questions were based on relevant components of two theoretical models: Social Cognitive Theory and the Health Belief Model [37,38]. Examples of questions from the mothers' interview guide included: What influenced your decision to breastfeed? What support from your partner have you found helpful during your breastfeeding experience? What support would you have liked to receive from your partner? Issues discussed with the fathers included questions such as: How important do you think breastfeeding is for a baby? How did you support your partner during her breastfeeding? Each focus group lasted from 60 to 90 minutes, and to minimise interviewer bias and ensure consistency, one of the research team experienced in focus group research facilitated all of the sessions.

All focus group discussions were recorded using two digital recorders, plus a research assistant wrote summary notes. Each participant signed a consent form prior to commencement of the focus groups or gave verbal consent for a telephone interview. Refreshments were provided to all participants attending the focus groups and a gift card incentive was given to every participant to acknowledge their time.

The online survey, developed through Survey Monkey, incorporated all the questions from the fathers' focus group and allowed for open ended questions. This type of questioning facilitated the collection of rich qualitative data.

### Data analysis

Qualitative data analysis consisted of two processes. The first involved identifying, coding, and categorising themes found in the data [39]. Data from the audio-tapes were transcribed by the first author and analysed using a constant comparison method modified from the grounded theory approach [40]. In this process, all transcribed data were coded manually and common themes and categories created. This created additional understanding of the interactions and perceptions of the participants. The participants' reflections, conveyed in their own words, strengthen the validity and credibility of the research [41]. Transcripts were cross-checked by four of the researchers and categories and themes corroborated to ensure credibility and conformability of the data analysis. The second process of analysis used the element of Social Cognitive Theory and the Health Belief Model as an analytical framework from which identified themes were considered.

## Results

The participants were mainly first time parents who were geographically located across the Perth metropolitan area. These locations included western (high income), eastern (average income), southern (lower income) and northern (average income) suburbs [42]. The age of mothers ranged from 18 to 37 years and fathers from 26 to 48 years. The majority of participants were married (70%, n = 53) or living in a defacto relationship (29%, n = 21), with two participants being single.

A number of practical support strategies were suggested by the mothers and included: assistance with meal preparation, housework such as washing dishes and/or clothes, shopping, bathing the baby, bringing the baby to the mother for a night-time feed and measures to assist the mother to relax, such as a neck massage. As far as providing emotional support, women suggested that their partner could give praise or compliments, plus boost her confidence with encouraging comments acknowledging her breastfeeding efforts.

Analysis of the data revealed two major themes, "Dads do make a difference" and "Wanting to be involved", relating to paternal support with sub-themes describing the perceptions of effective paternal support from both mothers and fathers. Mothers' responses identified three common sub-themes relating to fathers making a difference, which were: "Anticipating needs and getting the job done", giving "Encouragement to do your best" and having a "Paternal commitment to breastfeeding". Fathers' responses identified three common sub-themes related to being involved, which were: "Wanting relevant information", "Learning the role" and "Being an advocate".

Pseudonyms and participants age have been used to illustrate the participants' comments to support the themes developed.

### Mothers' major theme: Dads do make a difference

The theme "Dads do make a difference" emerged from all focus groups with a wide range of situations presented where fathers made a difference and numerous examples of practical ways in which they helped, including assistance with expressing breast milk:

"Without him I couldn't have done it [breastfeed], I couldn't have expressed for the two weeks that I did." (Melissa, age 27)

Similar thoughts were expressed by Camilla who acknowledged how much her partner supported her efforts when her family was pro formula feeding:

"None of my family has breastfed and they thought it was a step backwards. Only poor people breastfeed. They didn't understand what the urgency and importance of breastfeeding was for me. Without him [partner] I couldn't have done it [breastfed] really." (Camilla, age 22)

### Anticipating needs and getting the job done

Just being there to offer support as needed and sharing the new parenting burden was identified by many of the mothers as an important role. One mother spoke about how useful it was that her partner was able to remember the attachment and positioning skills for breastfeeding;

"He was sort of assisting me when the lactation consultant was around and he would remind me later, because I would forget things like that. He would actually observe and try to make suggestions about positioning and attachment, which was good for both practical and moral support."(Jane, age 28)

Mothers reflected on some of the ways their partners anticipated their needs and assisted them with their newborn babies:

"He just knew that that's what I needed; I didn't have to say the baby's awake, get up. He just got up straight away and brought her to me." (Sarah, age 33)

Carol offered examples of how her partner allowed her to take some time out:

"He'd come up and just hold the baby, so I could have a shower or grab something to eat." (Carol, age 32)

On occasions when mothers felt overwhelmed they relied on their partners to continue running the house:

"I was getting no sleep at that time; you know feeding her was taking two hours. Then there was an hour gap and I would have to start feeding her again, so he pretty much did everything; cooked, cleaned, went to work." (Pamela, age 29)

Another mother recognised the importance of her partner being home in the first few weeks post birth to help her.

"It was great to have him there during the day. I think it's really important that husbands need to be home each day for the first month to help, because that's when the problems tend to happen." (Marg, age 26)

### Encouragement to do your best

Offering the mother acknowledgement for the effort being put into breastfeeding and giving emotional support was seen to be especially important. Mothers talked about the difficulties in the first few weeks at home with a newborn, and how important it was that their partner was encouraging and accepted the time commitment to breastfeeding. As the following mother related:

"Just that encouragement, you know when your partner says you're doing a good job in those early days when you're feeding for 40 minutes or an hour, I found that really helpful." (Juanita, age 34)

One mother talked about her increased sense of confidence knowing her partner trusted her to do the best for their baby:

"It's like he trusts that I will do what is best for the baby." (Robin, age 23)

Acts of affection and kindness were greatly appreciated and acknowledged as this mother described:

"He got up during the night and gave me cuddles when I was in tears, and my nipples were cracked and sore and bleeding, that was really helpful." (Dina, age 21)

Another mother talked about the importance of her partner just being there, accepting of her commitment to breastfeed:

"He was fully aware that my job at that time was to feed the baby, and for him to just be there." (Rhani, age 30)

Anticipating needs, emotional support and a commitment from the father to support and promote breastfeeding, led to the third sub-theme.

### Paternal commitment to breastfeeding

Believing that breast milk was best for their child and willing to do what was necessary to assist his partner to breastfeed, saw one father go "head to head" with the hospital staff following his partner's emergency caesarean birth.

"Normally, he doesn't engage in discussions like this. But he'd taken it in and when it was necessary, he's stepped up and pushed back the medical staff and said to them 'breast milk is best. It's on tap. It's here, it's available, and that's what my baby's having.' And that meant the world to me. It's one of those situations where I could easily have come out of the general anaesthetic and gone. 'Crap, my baby's had formula for its first feed,' but it hadn't because Dad was on the ball, to say 'No'." (Julie, age 26)

Another mother spoke of the commitment and support from her husband to express breast milk to ensure their baby didn't have formula:

"She just wasn't a strong feeder, and I needed a lot of support; there was never any mention of formula or anything like that. He just knew to get the pump, and we pumped for six weeks." (Anna, age 32)

Using computer technology assisted the next father in his commitment to breastfeeding and reduced the attachment difficulties for his partner:

"Getting the attachment right was a bit challenging and I remember one night he went to our laptop computer, brought it into the bed and we watched a little demonstration on breastfeeding. He set that up for me, helping me get the attachment right." (Jacqui, age 22)

The mothers' experiences reflected how "Dads make a difference". However, when the fathers' data were analysed the major theme "Wanting to be involved" emerged. Three sub-themes also emerged: "Wanting relevant information"; "Learning the role" and "Being an advocate".

### Fathers' major theme: Wanting to be involved

Fathers participating in the study all wanted to be involved with parenting and parenthood, but many of them felt they were unprepared and lacked the relevant information to be effective in their parenting role. One father spoke about feeling left out and neglected because he didn't understand what his partner was going through:

"I felt like I was missing out on intellectual stimulation, missing out on emotional stimulation and physical. I think a lot of those things; the feeling of neglect could have been avoided by having someone explain to me what the women really go through and how to make things easier or better." (Imran, age 29)

Responses from fathers indicated there was a need for ready access to support specific to father's needs.

"Give some advice on how to be supportive to the mother when she is struggling with breastfeeding/baby blues." (Jason, age 22)

Fathers indicated they believed men may be reluctant to ask for help or not know who to ask or where to ask:

"Let fathers know why mothers need assistance and what they can do to help once the child comes along. Encourage them to get involved in day to day stuff." (Michael, age 32)

### Wanting relevant information

The men were consistent in identifying their need for "warts and all information".

"Information on relationship changes, hormonal changes of the partner and how best to support and handle those changes. Supporting your partner when she has problems like postnatal depression, no sex drive, changing hormones, crying/yelling/angry outbursts - learning to understand and support rather than chastise and think there is something wrong with your partner." (John, age 32)

Many of the fathers felt inadequate in their lack of knowledge about this new role in their lives and some felt resentful that they were not as informed as their partners. Even though fathers had often attended antenatal education classes they still felt inadequately prepared:

"You want similar information that mother's are given in mother's group on how to feed, nurture, and bond. Antenatal classes give the impression that fathers have nothing to do with their child." (Peter, age 38)

Most fathers believed they needed much more information about pregnancy, childbirth, breastfeeding and parenting. As Mark said:

"Provide information on why breastfeeding is best for baby and mother." (Mark, age 38)

Another father talked about the need for reassurance and support for fathers:

"It is easy to feel that the mother knows what to do and for the dad to stand back because he is a bit scared of doing it wrong. Confirmation that the mother is just as out of her depth as the father, and that it is a team effort." (Julio, age 25)

The following quote illustrates one father's need for information about difficulties associated with breastfeeding. His concern and horror about a piece of his wife's nipple falling off was in no way mediated by any of the information he had received during the antennal education process:

"Badly cracked nipples, one was so bad a small piece of the nipple fell off and left a hole, I didn't know what to do." (Muhammad, age 23)

Without exception, all the participating fathers wanted to be part of the parenting experience, but needed to learn the role.

### Learning the role

The requirement for pragmatic information and realistic solutions being incorporated into learning the role were identified by participants who talked with pride about their babies and what it meant to be a father. As one dad said:

"Watching him develop, having cuddles, anticipating of our future together. Seeing part of yourself in your little boy. Showing him off to family, friends and everyone else. Seeing him smile. Watching him interact with his beautiful mother." (Rick, age 37)

Many of the fathers had ideas about what would have been useful for them to know before they had the baby. As the following father related:

"How to support your partner, things you can do to be involved. How to comfort your partner, the kind words you can say to support her. Hints on helping and understanding new mothers. Some advise on caring for the new baby." (John, age 27)

One father talked about having a realistic view of what to expect: and just being there as a comfort for his partner: "A no bullshit idea of what to expect and how to help even if that means doing nothing but being there with her and the baby." (Pete, age 28)

### Being a breastfeeding advocate

Fathers discussed the need to advocate to family members and health professionals on the importance of their baby being breastfed.

"It is actually a sacrifice but at the same time, people should realize that if you feed baby properly [breastfeed] you will have your life more comfortable than if you don't. When baby's happy then everything is good." (Andre, age 37)

To be there and protect and defend parenting decisions against negative or unhelpful interference such as extended family who encouraged formula or undermined the mother's efforts was very important for fathers. As this father explained to his extended family:

"This is our parenting journey. Please be respectful, we feel it's best to do it this way [breastfeed]. Thank you for understanding." (Aaron, age 36)

Another father spoke about the advocacy role he played when the midwives at the hospital wanted to give his baby formula, knowing his partner wanted to breastfeed:

"I know you want to breastfeed, so stick to your guns." (Jarrad, age 40)

Supporting the decision to breastfeed in public without feeling shame was highlighted by the following father's comments. He also describes the shift from a sexual to functional use of the breast:

"When you're out and about sometimes it can be little bit concerning for a new mum to you know, just hang it all out. I guess there's still that well, it's like shame, and you don't want everyone looking at things [breasts] that have been private. And suddenly you've gone from being a sexual thing to a kitchen utensil." (Eric, age 38)

## Discussion

Acknowledging the inherent limitations of this research (non-representative self-selected sample, small sample size), the researchers were still able to access 76 parents from a range of socioeconomic settings, and were able to identify several consistent themes. While it was difficult to recruit fathers for focus groups due to time constraints associated with work commitments, fathers responded to an online survey that they could do in their own time. This data collection process enabled fathers to express their fears and concerns about the whole process of parenting and their lack of adequate information and preparation.

There was a consistency between the themes that emerged from both the mothers and the fathers, with both believing that breastfeeding was a team effort and that father's support was essential to the mother being able to breastfeed successfully. The tasks and activities fathers provided, as identified by the mothers, such as encouragement and problem solving were consistent with the requirements that fathers identified; for example the need for real information. In support of these themes, Wolfberg et al. found that breastfeeding initiation rates were higher (74%) when fathers attended a two hour prenatal intervention than in a control group (41%) [43].

In acknowledging the importance of paternal support for successful breastfeeding, Susin and Giugliani found that mothers would like more help from their partners, but were sometimes unclear what type of help they wished to receive [44]. They also found most fathers wanted to help mothers but did not know what they could do to help, which again reflects the fathers' views in the sub-theme "Learning the role". The sub-themes of "Encouragement to do your best" and "Being an advocate" were supported in the literature by Scott et al [45] and Scott and Binns [46], who identified the father as the most important support person to give encouragement and advocacy.

Barriers to effective breastfeeding identified by the participants included physical problems (such as poor attachment and cracked or bleeding nipples) contributing to challenges with breastfeeding, inadequate knowledge about how to manage potential breastfeeding problems, and exclusion of the male partner during the antenatal classes. Disturbingly, fathers identified a lack of "real information", a lack of recognition for their role, lack of engagement during the antenatal education process, and lack of commitment to breastfeeding by hospital staff as barriers they needed to overcome in their role as advocates and supporters of breastfeeding. This finding is supported by two recent studies: Pisacane et al. [47] found that supporting fathers with information about the breastfeeding process increased the duration of breastfeeding, and Rempel and Rempel [48] found it was important to provide men with the evidence supporting breastfeeding so that they had a solid basis on which to develop pro-breastfeeding beliefs. The need for clear concise information is essential if fathers are to be advocates for breastfeeding.

The findings from this study highlight the importance of practical, emotional and physical support for mothers. Research suggests many women have difficulty breastfeeding and need the support of their partner to be successful. The importance of breastfeeding was identified by the fathers in this study. Giving fathers more information about the breastfeeding benefits for both mother and baby [47], and possible problems associated with breastfeeding can give them the confidence to support their partners and become breastfeeding advocates [49,50]. Similarly, Sheehan et al. suggest fathers not only influence the decision to breastfeed, but they also play an instrumental role in whether mothers continue breastfeeding or stop prematurely [51]. Ingram and Johnson worked with fathers to increase breastfeeding support for mothers and found that fathers' attitudes to breastfeeding in public and knowing how much milk the baby was getting had the most influence on whether they supported their partner to continue to breastfeed [52].

The men participating in this study clearly wanted information about how they could support their partners in the postnatal period including support of breastfeeding, and were motivated and ready to learn. Teaching fathers how to prevent and to manage the most common lactation difficulties is associated with higher rates of breastfeeding at six months [47]. Breastfeeding cannot be promoted without it also being supported socially, economically, and politically [53], and women should be encouraged to breastfeed, while institutions and communities are challenged to remove the barriers to breastfeeding continuation [54]. Identifying the different methods of support can assist antenatal educators to promote the skills necessary to successfully breastfeed.

There was disparity between the advice and support some health professionals provided and the needs of the fathers as breastfeeding champions in this study. This was also found by Hauck et al., who found conflicting advice from health professionals gave reduced support for mothers to breastfeed [24]. This study strongly supports policy changes within the maternity units that reflect a commitment to breastfeeding and that reduce conflicting advice. Recommendations that health professionals should educate all key family members, both during pregnancy and in the postnatal period, on the benefits of breast milk, and on how to encourage and support mothers in the early weeks of breastfeeding have already been made [55,56]. A move towards Baby Friendly hospitals across the state would greatly increase the opportunity for greater education and support for ongoing breastfeeding as the natural choice for infant feeding. Increasing Baby Friendly hospitals globally could increase both breastfeeding initiation and duration, and this study promotes the continuance of these recommendations.

## Conclusion

Men want to be part of the parenting role and need information and knowledge. This would give them the opportunity to synthesise the information and apply the knowledge to feel confident and competent in their new role as an involved parent.

The role of practical and emotional support from fathers is an essential ingredient to successful breastfeeding, increasing the mother's confidence and enabling her to maintain an adequate milk supply. Whilst breastfeeding remains the sole domain of women, the essential support of their partners can be a lost opportunity. Empowering both parents to make and sustain a commitment to breastfeeding requires the infrastructure and human resources to make this possible. The fact that some participants in this study experienced a lack of commitment to breastfeeding by some hospital staff may indicate that additional training is required in hospitals. Baby Friendly hospitals Australia-wide and globally could be the first step to educating maternity staff and health professionals to the importance and benefits of breastfeeding, though if Bartington et al.'s findings are correct, perhaps an alternative to hospital-based education needs to be found, one that engages fathers as well as mothers [16]. However, recognition that breastfeeding is a family issue benefits everyone. When difficulties encountered by mothers are shared with their partners, babies will have a better chance of receiving breast milk exclusively for the recommended six months, and with complementary food could continue to breastfeed for two years or more.

## Competing interests

The authors declare that they have no competing interests.

## Authors' contributions

JT participated in the data collection and analysis and drafted the manuscript. BM has made substantial contributions to conception and design and in revising the manuscript for intellectual content and given final approval. YLH has made substantial contributions to conception and design, acquisition of data and analysis and interpretation of data; involved in manuscript revision. PH has made substantial contributions to conception and design, acquisition of data and analysis and interpretation of data; involved in manuscript revision. SB has made substantial contributions to conception and design and participated in manuscript revision. CWB has made substantial contributions to conception and design. All authors read and approved the final manuscript.
